# Measuring the Potential Impact of Combination HIV Prevention in Sub-Saharan Africa

**DOI:** 10.1097/MD.0000000000001453

**Published:** 2015-09-18

**Authors:** Amin Khademi, Sunanth Anand, David Potts

**Affiliations:** From the Department of Industrial Engineering, Clemson University, Clemson (AK, SA); and Anmed Health Medical Center, Anderson, South Carolina, USA (DP).

## Abstract

A public health approach to combination HIV prevention is advocated to contain the epidemic in sub-Saharan Africa. We explore the implications of universal access to treatment along with HIV education scale-up in the region. We develop an HIV transmission model to investigate the impacts of universal access to treatment, as well as an analytical framework to estimate the effects of HIV education scale-up on the epidemic. We calibrate the model with data from South Africa and simulate the impacts of universal access to treatment along with HIV education scale-up on prevalence, incidence, and HIV-related deaths over a course of 15 years. Our results show that the impact of combined interventions is significantly larger than the summation of individual intervention impacts (super-additive property). The combined strategy of universal access to treatment and HIV education scale-up decreases the incidence rate by 74% over the course of 15 years, whereas universal access to treatment and HIV education scale up will separately decrease that by 43% and 8%, respectively. Combination HIV prevention could be notably effective in transforming HIV epidemic to a low-level endemicity. Our results suggest that in designing effective combination prevention in sub-Saharan Africa, priorities should be given to achieving universal access to treatment as quickly as possible and improving compliance to condom use.

## Abstract 2

Supplemental Digital Content is available in the text

## INTRODUCTION

HIV/AIDS is one of the world's pressing infectious diseases. More than 35 million people are infected with HIV and this number is still increasing.^1^ The area hit hardest by the epidemic is sub-Saharan Africa where more than 22 million are infected and the primary route of infection is heterosexual.^1^ Significant efforts have been carried out by sub-Saharan African nations, in cooperation with developed nations, the World Health Organization (WHO), the pharmaceutical industry, and many private charities to curb the epidemic. A public health approach to combination HIV prevention is recently advocated to transform HIV transmission from a pandemic to low-level endemicity.^2^ The purpose of this study is to develop a dynamic HIV transmission model to measure the impacts of a combined HIV prevention program on HIV trends in sub-Saharan Africa. In particular, we consider universal access to treatment (defined as coverage of at least 80% of the population in need) combined with HIV education scale-up.

HIV prevention programs have been quite successful in sub-Saharan Africa. The availability of antiretroviral therapy (ART), the only treatment option for chronic HIV, has substantially increased over the last decade in sub-Saharan Africa: treatment coverage has increased from 3% in 2003 to 50% in 2013.^3^ Moreover, HIV education that is a key to every HIV prevention program has been highly promoted and many countries in the region have developed national policy on HIV/AIDS education.^4^ For instance, the ABC strategy (abstinent, be faithful, use a condom) to HIV prevention decreased the HIV infection rate in Uganda from 15% in 1991 to 5% in mid-1990s.^5^ Partner reduction and fidelity has had a significant impact on incidence reduction in several parts of the region. In Cambodia where the prevention efforts reduced HIV incidence, the proportion of men who reported paying for sex has declined.^6^ In Zambia, faith-based campaigns to promote abstinence and monogamy among young people decreased the HIV prevalence in young women during the 1990s.^7^ A decline in HIV incidence in Kenya is reported to be associated with behavioral changes.^8^ Similar trends are observed in Addis Ababa, Ethiopia, and Dominican Republic.^9^

Modeling the impacts of ART on HIV progression in an HIV-infected individual, as well as a population of susceptible and infected individuals, has received a significant attention in both resource-rich and resource-limited settings. In the patient level, Perelson et al^10^ examined a model for the interaction of HIV with CD4 cells and considered the effects of AZT on viral growth and CD4 dynamics. Braithwaite et al^11^ developed a simulation model for HIV progression in an infected individual and examined alternative thresholds for ART initiation. Walensky et al^12^ created a simulation model to inform HIV treatment decisions regarding the optimal CD4 threshold in South Africa. In the population level, researchers have developed models to investigate the consequences of ART scale-up on the HIV-infected population.^13–17^ In particular, Granich et al^13^ developed a mathematical model that predicted that HIV can be eliminated in South Africa by implementing the test-and-treat strategy in 40 years with approximately $10 billion less cost than universal access to treatment.

Despite significant research on modeling the impacts of ART on HIV trends, the literature on modeling the potential impacts of HIV education scale-up on HIV trends is scarce and most attention is given to designing clinical studies to investigate the effects of behavioral change in the population on HIV progression.^18–21^ Enns et al^22^ created a model to estimate the effectiveness of changes in concurrent sexual partnerships in reducing the spread of HIV in sub-Saharan Africa. Kretzschmar and Morris^23^ investigated the impact of concurrent partnerships on epidemic spread. Kessler et al^24^ estimated the reduction in HIV incidence in New York City due to behavioral changes via simulation. However, the focus of these studies is on modeling one of the benefits of HIV education at a time and they did not model the simultaneous effect of various benefits of HIV education. In this study, we develop an analytical framework to estimate the effects of HIV education on HIV metrics such as incidence, prevalence, and mortality. In particular, we consider compliance to partner reduction and condom use and our results shed light on the role compliance plays in curbing the epidemic.

## METHODS

In a broad view, we classify the population into 3 categories: sexually active susceptible individuals (hereafter, susceptible individuals), infected individuals not on treatment, and infected individuals on treatment. Let X(t), Y(t), and Z(t) denote the total number of susceptible, nontreated infected, and treated infected individuals, respectively. To be able to model viral loads (hence infectivity), we classify infected individuals into 3 groups: primary, chronic, and symptomatic stages of HIV infection.^25^ That is, *Y*_1_(*t*), *Y*_2_(*t*), and *Y*_3_(*t*) denote the number of untreated infected individuals in primary, chronic, and symptomatic stage of HIV at time *t*, respectively. Therefore, infectivity is highest in primary infection, lower in chronic infection, and increases again in symptomatic stage. We assume that HIV-infected individuals spend ∼2 months in primary infection, ∼7.5 years in the chronic stage, and ∼3.5 years in the symptomatic stage.^25^ Also, let *Z*_1_(*t*), *Z*_2_(*t*), and *Z*_3_(*t*), respectively, denote the number of treated infected individuals in primary, chronic, and symptomatic stage of HIV at time *t*. We use data available in the literature to estimate the survival time of infected individuals after initiating treatment.^25^ At time *t* the total number of individuals who become sexually active and join the susceptible population is denoted by *b*(*t*). Let *I*(*t*) and *P*(*t*) be the incidence and prevalence of the disease at time *t*. We assume that the transmission is due to heterosexual partnership. This assumption is mild in our setting because concurrent sexual partnership is the key driver in HIV epidemic in sub-Saharan Africa.^26^

**FIGURE 1:**
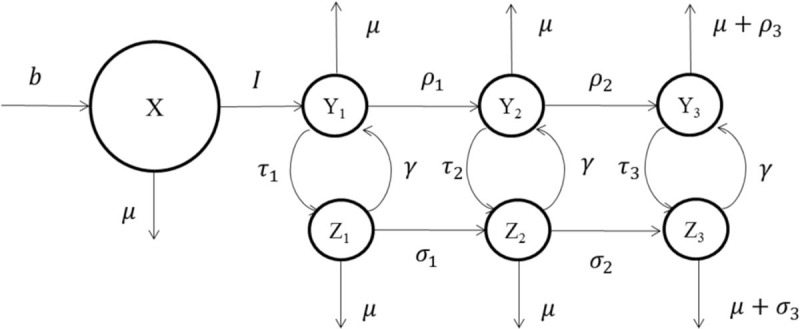
Model overview. See Appendix 1, http://links.lww.com/MD/A393 for details regarding the transmission model.

Aligned with the literature, we consider an exponentially decay function to incorporate the heterogeneity in the population. In particular, we use λe^−αP(*t*)^, where the transmission rate takes value of *λ* (transmission parameter) in the beginning of the epidemic and decays exponentially with rate *α* times the prevalence.^27^ Therefore, we have 
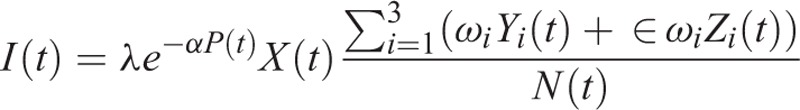


where *ω*_*i*_ denotes the infectivity of an infected individual in category *i* per unit time, ∈ captures the effect of treatment on reducing the infectivity of an infected individual, and *N*(*t*) denotes the total number of individuals in the population. Infected individuals on treatment may stop using treatment due to side effects of ART or they may develop resistant mutations. In order to incorporate these phenomena, infected individuals with rate *γ* move from being on treatment to not on treatment. The underlying assumption is that the health progression of infected individuals after stopping treatment or developing resistant mutations is similar to untreated individuals (see Granich et al^13^ for this assumption). Figure 1 shows an overview of the model and Table 1 provides a complete list of parameters used in the model as well as their values. See Appendix 1, http://links.lww.com/MD/A393, for more details regarding the transmission model.

**TABLE 1:**
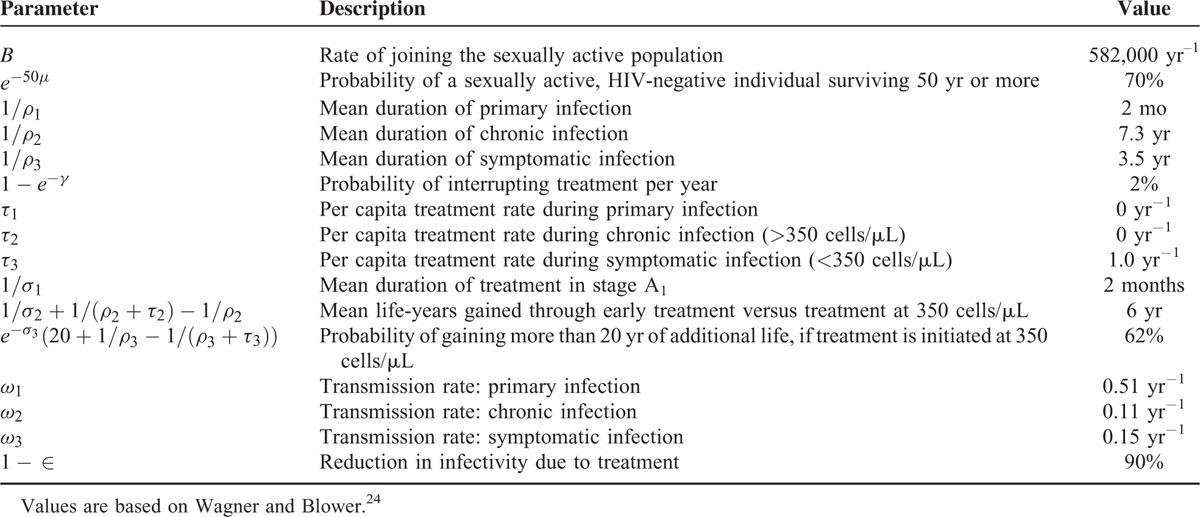
Model Parameters and Their Values

In our model, HIV education affects the epidemic by changing the sexual behavior of individuals in 2 dimensions: the expected number of partners that an individual establishes, and the likelihood of using condoms. Although HIV education has potentially other benefits such as stigma reduction and HIV test rate increase, we considered the 2 abovementioned factors because studies show that most benefit of HIV education is due to a reduction in partner acquisition and an increase in the frequency of condom use.^8^ Next, we show how HIV education impacts incidence. The transmission parameter, *λ*, can be written as 
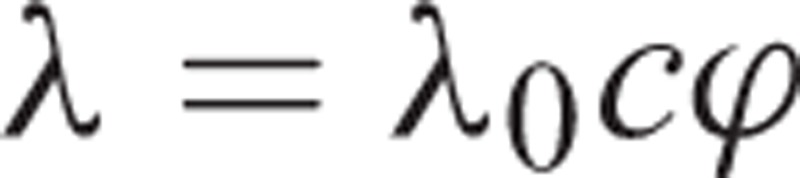


where *c* is the number of partners that a susceptible individual establishes in a period, and ϕ is the probability of infection in a partnership.^28^ As mentioned earlier, education impacts both *c* and ϕ, that is, both of them are a function of education. Let 0 ≤ θ  ≤ 1 denote the proportion of individuals in the society educated with sexually transmitted diseases (STD), where zero shows that nobody is STD educated and one shows that all individuals are STD educated. We consider the compliance of individuals to partner acquisition reduction and condom use in our model. Let ϑ_1_ and ϑ_2_denote the compliance of an STD-educated individual to partner acquisition reduction and condom use, respectively. Note that ϑ_1_ and ϑ_2_may depend on other factors such as time and education level, but for ease of notation, we drop any dependences. By assuming a linear relation, we have 
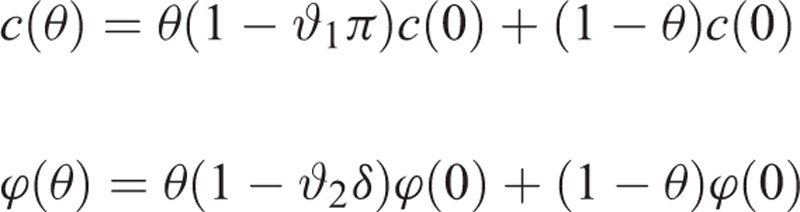


where *π* is the reduction in number of partners due to education, and *δ* is the reduction in the probability of infection due to condom use. Now suppose that we increase the level of education in the society and the new proportion of STD-educated individuals is *θ′* ≥ θ. A similar calculation reveals that 
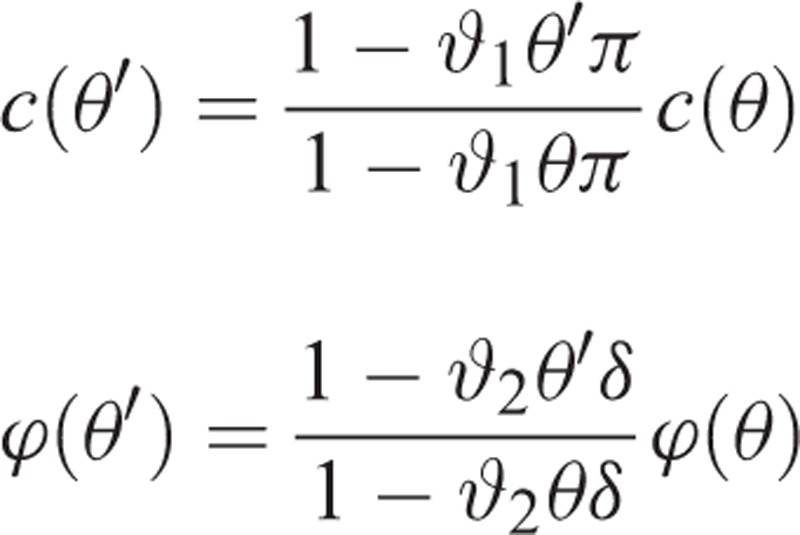
.

Therefore, the effect of education scale-up on incidence is given by  
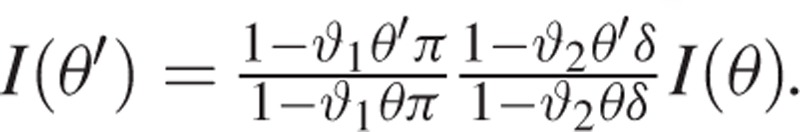
.

We used demographic and epidemiologic data from South Africa to parameterize our model. Then, we calibrated the transmission model by comparing the HIV prevalence generated by the model with the actual HIV prevalence observed in South Africa from 1990 to 2000 because ART was insufficiently available during that period.^29^ Pursuant to this goal, we set the initial state of the epidemic in the model to that observed in 1990 in South Africa and change the heterogeneity factor, *α*, to fit the curve.

We used the calibrated model to simulate the impacts of a variety of intervention combinations on the epidemic. In particular, we simulated the impacts of HIV education scale-up and universal access to treatment separately as well as a variety of their combinations. For simulating universal access to treatment, we started treating infected individuals as soon as they moved to the symptomatic stage. We estimated the HIV education parameters by using the data available in the literature. In particular, we assumed that STD-educated individuals have 35% reduction in number of partners and their compliance to partner reduction is 77%.^30^ We assumed that condom use will decrease the likelihood of infection by 90% and the compliance of STD-educated individuals to condom use is 59%.^31,32^ Because we could not locate a comprehensive study on HIV literacy rate in sub-Saharan Africa, we used the literacy rate as an indicator of HIV literacy rate as they are strongly correlated.^33^ Therefore, we assumed that the HIV literacy rate is 66%.^34^ We increased the HIV literacy rate to 90% in the population and simulated its impact on the epidemic. We also conducted several sensitivity analyses to test the robustness of the results by changing the values of model parameters.

Since implementing universal access to treatment and HIV education scale-up will take time in practice, we assume that their coverages increase logistically in time. That is, if we let θ(t) be the HIV literacy rate at time *t* and θ¯ be our target for the HIV literacy rate, we use the following formula: 
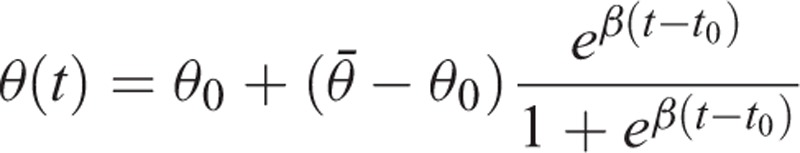


where *θ*_0_ is the initial literacy rate, *t*_0_ is the time at which the logistic term reaches 0.5, and *β* determines the rate at which the literacy rate increases. We set these parameters in the model such that the target is reached within 2 years. A similar approach is used for increasing the treatment coverage over time. Note that because this is a modeling study, ethical approval is not necessary.

## RESULTS

**FIGURE 2:**
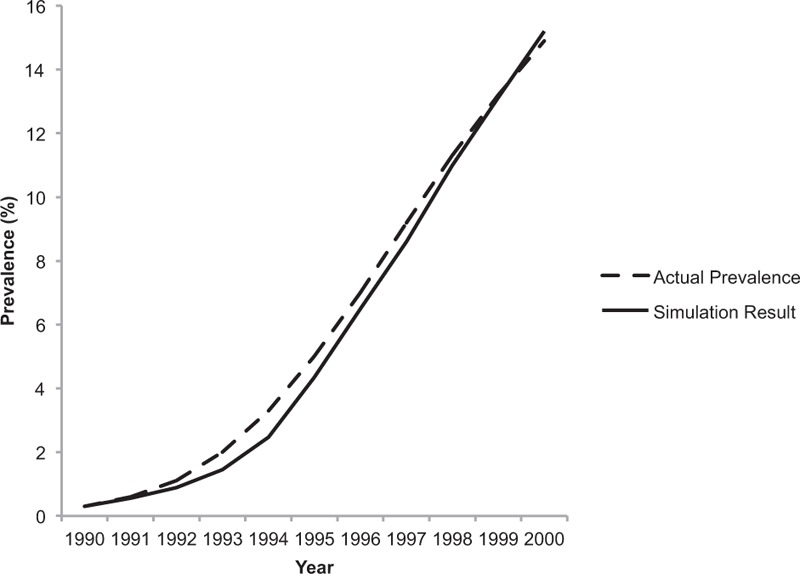
Model calibration. It shows the result of the calibration phase. We used demographic and epidemiologic data from South Africa to parameterize our model. It compares the HIV prevalence generated by the model (solid line) with the historical prevalence (dotted line) observed in South Africa from 1990 to 2000.

**FIGURE 3:**
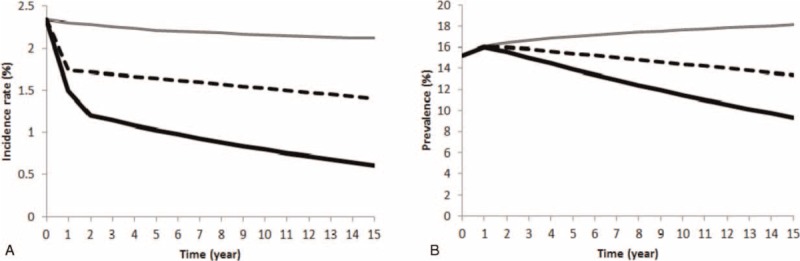
Results of simulation. Panels (A) and (B) show the evolution of HIV incidence rate and prevalence under different strategies over the course of 15 years, respectively. Our results show that by just implementing HIV education scale-up strategy (compound line), the incidence rate over 15 years drops from 2.3% to 2.1%; thus prevalence keeps increasing. Implementing universal access to treatment (dotted line) decreases the incidence rate from 2.3% to 1.3% in 15 years and consequently prevalence decreases from 15.1% to 13.3% over this period. Universal access to treatment combined with HIV education scale-up significantly decreases the incidence rate from 2.3% to 0.6% which results in a substantial prevalence drop of ∼6% over 15 years.

Figure 2 shows the results of the model calibration. It compares the HIV prevalence generated by the model (solid line) with the historical prevalence (dotted line) observed in South Africa from 1990 to 2000. We used the calibrated model to investigate the impact of combination of various interventions on the epidemic for 15 years. Figure 3 shows the results of the simulation in terms of incidence rate and prevalence for HIV education scale-up (compound line), universal access to treatment (dotted line), and HIV education scale-up along with universal access to treatment (solid line).

Our results show that by just HIV education scale-up, the epidemic growth slows down and the incidence rate slightly drops. During the course of 15 years, the incidence rate changes from 2.3% to 2.1%. By implementing universal access to treatment, our results show that the incidence rate drops and decreases over time which results in a decrease in prevalence. In particular, the incidence rate changes from 2.3% to 1.3% and the prevalence changes from 15.1% to 13.3%. More importantly, our results show that HIV education scale-up combined with universal access to treatment significantly decreases the incidence rate from 2.3% to 0.6% and prevalence decreases from 15.1% to 9.3% over a course of 15 years. This shows that the benefit of a combined strategy of universal access to treatment and HIV education scale-up is greater than the summation of their individual benefits, that is, it has super-additive property. In particular, the combined strategy will decrease the incidence rate by 74% over the course of 15 years, whereas universal access to treatment and HIV education scale-up will separately decrease that by 43% and 8%, respectively.

We observed a reduction in the cumulative HIV-related deaths by implementing the interventions. Compared with HIV education scale-up strategy, universal access to treatment averted 7,596,439 deaths whereas universal access to treatment along with HIV education scale-up averted 7,679,917 deaths in the course of 15 years. This shows that the significant reduction in HIV-related deaths is due to universal access to treatment.

We conducted a sensitivity analysis to test the robustness of model results and to investigate the significance of each parameter on model outcomes. We perturbed the model parameters for the combined strategy of universal access to treatment along with HIV education scale-up. Table 2 reports the results of the HIV incidence rate reduction after 15 years compared with the initial incidence rate, and the cumulative number of HIV-related deaths averted compared with that in the HIV education scale-up strategy. Our results show that by considering the current reports on model parameters in the literature, compliance to condom use is the most critical factor: if it dropped to 30%, the incidence rate reduction would decline to 60% (for the base case it is 74%), and if it increased to 90%, the incidence rate reduction would increase to 87%. This insight suggests that efforts in designing more effective HIV education programs should be on promoting individuals’ compliance to condom use.

**TABLE 2:**
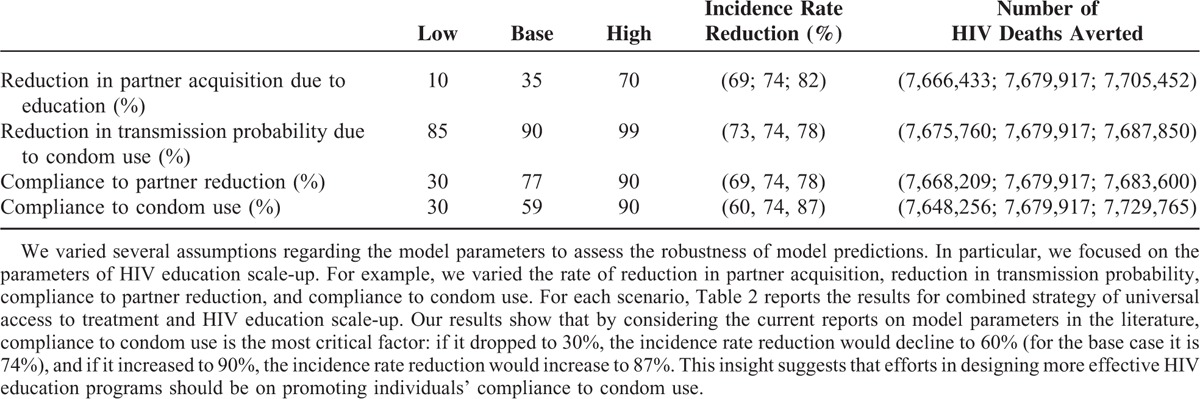
Sensitivity Analysis

## DISCUSSION

HIV prevention programs that aim to stop the spread of the disease have been relatively successful in reducing the incidence rate in sub-Saharan Africa during the past 2 decades. Recently, more comprehensive prevention strategies are available due to scientific and epidemiological advances. In particular, combination prevention is highly advocated to achieve maximum effect on curbing the epidemic in a specific setting. In this study, we developed a dynamic HIV transmission model to estimate the impact of simultaneous implementation of universal access to treatment and HIV education scale-up in sub-Saharan Africa. We assumed that HIV education impacts the behavior of individuals in reducing the number of sexual partners and in increasing the likelihood of condom use. We did not consider other potential benefits of HIV education scale-up such as HIV test rate increase and stigma reduction. Therefore, our results underestimate the benefits of HIV education scale-up on the epidemic.

We calibrated the model with data from South Africa and tested the impact of a variety of intervention combinations on the epidemic. Our results show that incidence reduction by implementing universal access to treatment along with HIV education scale-up is substantially larger than the summation of incidence rate reduction of universal access to treatment and HIV education scale-up separately. This observation confirms that comprehensive combination prevention might have a larger impact on containing the epidemic than implementing disperse prevention. Moreover, our sensitivity analyses show that compliance to condom use plays a key role in controlling the epidemic. This observation suggests that for designing an effective HIV education program, policy makers may prioritize funding to educational programs that aim to improve the compliance of individuals to condom use.

This study has several limitations. We did not consider different risk groups such as men who have sex with men, injecting drug users, and sex workers in the model. Also, we did not consider gender in the model although HIV education may disproportionately affect men and women. However, the results of this simple and parsimonious model show that behavioral changes have a significant impact on the epidemic and changing the behavior of riskier individuals would probably have a larger impact than that reported in this work. We did not model the development of drug resistance on treatment and as a result the model does not consider the evolution of acquired resistance and the dynamics of transmitted resistance. Cost or cost-effectiveness has not been considered in our analyses, which may have impact on policy recommendations. In summary, our results suggest that a public health approach to combination HIV prevention may transform the HIV transmission from a pandemic to low-level endemicity, and shed light on the role behavioral change and treatment play in containing the epidemic.
